# Failure Analysis of Gear on Rail Transit

**DOI:** 10.3390/ma18204773

**Published:** 2025-10-18

**Authors:** An-Xia Pan, Chao Wen, Haoyu Wang, Ping Tao, Xuedong Liu, Yi Gong, Zhen-Guo Yang

**Affiliations:** 1 School of Mechanical Engineering and Rail Transit, Changzhou University, Changzhou 213164, China; 2 Jiangsu Key Laboratory of Green Process Equipment, Changzhou University, Changzhou 213164, China; 3 Institute of Materials Modification and Modelling, School of Materials Science and Engineering, Shanghai Jiao Tong University, Shanghai 200240, China; 4 Shanghai Key Laboratory of Materials Laser Processing and Modification, Shanghai Jiao Tong University, Shanghai 200240, China; 5 CRRC Qishuyan Institute Co., Ltd., Changzhou 213011, China; 6 Department of Materials Science, Fudan University, Shanghai 200433, China

**Keywords:** high-speed train, gear, failure analysis, slag inclusions, grinding, cracking

## Abstract

The gear transmission system is a safety-critical component in rail transit, typically designed for a service life exceeding 20 years. Failure analysis of such systems remains a key focus for railway engineers. This study systematically investigates four representative cases of premature gear failure in high-speed trains using a standardized analytical procedure that includes visual inspection, chemical analysis, metallographic examination, scanning electron microscopy, and hardness testing. The results identify four primary root causes: subsurface slag inclusions in raw materials, inadequate heat treatment leading to a non-martensitic layer (∼60 μm) at the tooth root, grinding-induced temper burns (crescent-shaped "black spots") accompanied by a hardness drop of ∼100–150 HV, and insufficient lubrication. The interdependencies between these factors and failure mechanisms, e.g., fatigue cracking, spalling, and thermal scuffing, are analyzed. This work provides an evidence-based framework for improving gear reliability and proposes targeted countermeasures, such as ultrasonic inclusion screening and real-time grinding temperature control, to extend operational lifespans.

## 1. Introduction

The rapid expansion of rail transit infrastructure in China has yielded remarkable achievements in both network mileage and construction scale, significantly alleviating urban traffic congestion and reducing environmental pollution, thereby generating substantial socio-economic benefits [1,2]. Within this context, the gear transmission system serves as a critical component for power transfer in railway trains, transmitting torque from the traction motor to the wheelset. Compared to conventional industrial gears, railway gears operate under more demanding conditions—characterized by high rotational speeds, significant load shocks, and a broad operating temperature range—making them susceptible to unexpected failures [3,4]. Consequently, failure analysis of these components has become a major focus for railway engineers.

Gears are often subjected to cyclic loads; so, fatigue failure is one of the most important failure forms [5,6]. Some researchers have investigated the different causes of tooth cracks on gears. Zheng et al. [7] investigated the cause of premature failure of a 20MnCr5S gear. According to their analysis, the unqualified heat treatment process is the main reason for the fracture. The formed bainite accelerated the crack propagation, and finally, intergranular fracture and cleavage fracture occurred. Zhang et al. [8] analyzed the reason for the fatigue failure of a 20MnCr5 gear; insufficient carburization resulted in lower hardness, and the carburized layer depth was not up to standard. In the subsequent work, they optimized the carburizing process, which greatly improved the fatigue strength and life of the carburized gear. A helical gear pinion failed shortly after service; Ghosh et al. [9] identified the root cause of failure, and a metallography evaluation indicated a significant distribution of non-metallic inclusions near the fracture initiation region, causing the initiation of cracks. Fracture phenomena caused by inclusions and heat treatment processes were also reported by Cai et al. [10]. Qin et al. [11] conducted a failure analysis on the internal teeth of the ring gear used in the reducer of a coal mining machine. The study results showed that non-metallic inclusions, machining tracks, network nitride, and highly brittle microstructures were the main failure causes. Park et al. [12] carried out a comprehensive inspection of a defective planetary gear; the obvious fatigue stripes were observed by a scanning electron microscope, combined with finite element analysis. They concluded that the failure was caused by the blowhole that affected the fatigue fracture of the planetary gear. Zhang et al. [13] performed a series of examinations to identify the root cause of the straight bevel gear. After geometrical examination, differences were found in the tooth back thickness and tooth root fillet radius between the failed gears and intact gears, and subsequent research showed that the machining error caused excessive stress concentration at the root fillet, which led to fatigue cracks. During the analysis of a failed planetary gear in a windmill gear system, Rajinikanth [14] showed that the failure was initiated on the gear surface in the form of micro-pitting, which aided the spalling, propagation of an elliptical crack path, and gear tooth fracture. Pan et al. [15] analyzed the premature cracking of the driven gear of a high-speed railway gearbox; the results showed that the failure mechanism of the gear was rolling contact fatigue (RCF), which was mainly caused by the reduced hardness and contact fatigue strength of the meshing surface from grinding burn due to an improper grinding process. In addition, some scholars are committed to using numerical analysis to simulate crack propagation after initiation. In part of their research, crack tip stress intensity, rim thickness, and initial crack location [16] are important factors affecting the crack propagation path. Chen et al. [17] considered the possible influence of the initial angle on crack propagation and failure behavior. Doğan et al. [18] investigated the effect of rim thickness and drive side pressure angle on gear tooth root stress and fatigue crack propagation life. With the crack extension, there will eventually be a tendency to break the teeth and damage the gear body.

As evidenced by the literature, gear failures are often multifactorial, involving interrelated issues in raw material quality, heat treatment, manufacturing processes, and operational conditions. Therefore, systematic failure analysis is essential for enhancing gear reliability. It enables (i) the identification of root causes and evaluation of corrective measures to improve product quality; (ii) the assessment of design rationality and detection of potential flaws; (iii) the detection of deficiencies in cold and hot working processes to optimize manufacturing techniques; and (iv) the verification of material suitability and raw material quality.

This study analyzes representative gear failure cases in rail transit systems, examining influencing factors across four key categories: raw materials, heat treatment, manufacturing processes, and operational conditions. Based on the findings, practical preventive measures are proposed to mitigate the risk of premature gear failure.

## 2. Failure Case Analysis and Discussion

The following sections present the analysis and discussion of four distinct failure cases, examining the respective roles of raw materials, heat treatment, manufacturing processes, and service factors. A systematic failure analysis procedure was conducted for each case to ensure a comprehensive and accurate diagnosis of the root causes. The general investigative workflow, applied to all gears, followed a sequential protocol: it commenced with a macroscopic visual examination of the failed components to document the failure morphology and identify potential crack initiation zones. This was followed by chemical composition analysis using spark spectrometry to verify the material grade conformity. Critical sections containing cracks and failure origins were then excised for analysis. Metallographic samples were prepared, mounted, and etched for microscopic examination. Scanning electron microscopy (SEM), coupled with energy dispersive spectroscopy (EDS) (equipment is Hitachi S-3700N Scanning Electron Microscope, Japan), was employed to characterize the fracture surface morphology, identify microstructural features, and analyze the chemical nature of inclusions. Finally, microhardness profiles were measured using a Vickers hardness tester (equipment is HV-1000, China) from the surface to the core to evaluate the effectiveness of heat treatment and identify any anomalies, such as softening due to grinding burns. This standardized methodology ensures the transparency and reproducibility of our investigative process.

### 2.1. Effects of Raw Materials

Generally, gear manufacturing enterprises in the rail transit industry purchase the forged billet directly as the raw material for subsequent processing and manufacturing. The effects of raw materials at the present stage mainly include the following aspects.

#### 2.1.1. Purity of Raw Materials

**Figure 1:**
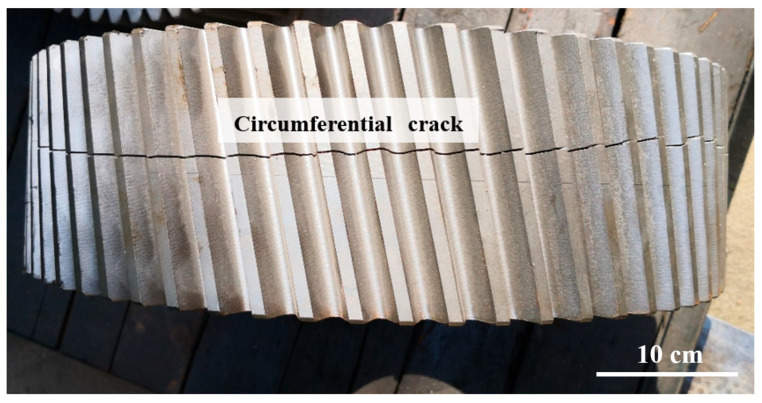
Digital camera photo of gear shaft with quench cracking.

Figure 1 shows the digital camera photo of a model locomotive gear shaft, which is made from the material 18CrNiMo7-6; the surface is heat-treated with carburizing and quenching processes. However, when the gear is finally quenched, a circumferential crack appears, and the crack penetrates all gear teeth. Interestingly, cracks do not occur at stress concentration positions, such as tooth roots or transition fillets. Through wire cutting, the gear is dissected along the crack so that the crack can be opened manually and more fracture characteristics can be observed.

**Figure 2:**
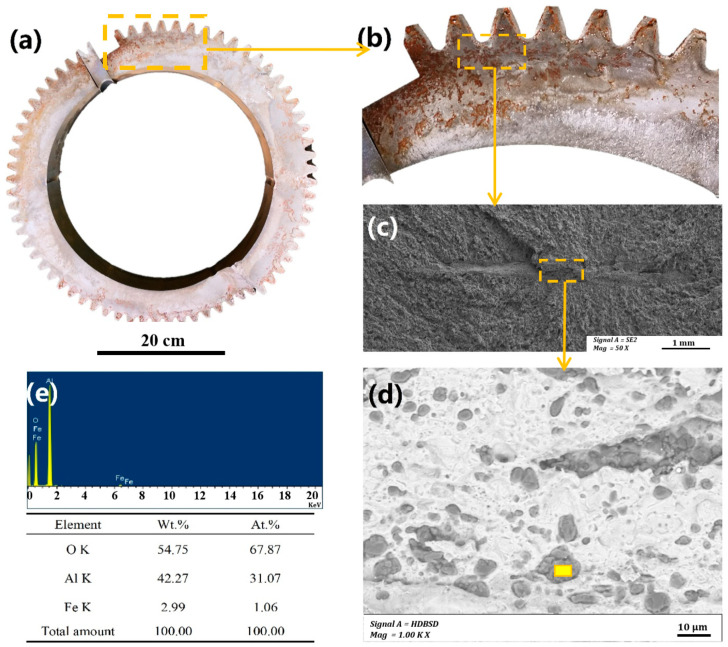
Failure overview of the gear: (**a**) appearance of the failed gear, (**b**) crack source, (**c**) micro-morphology of slag inclusion, (**d**) BSE image, and (**e**) EDS pattern of slag inclusion at the yellow mark in (**d**).

Figure 2a shows a digital camera image of the gear. The radial direction of the stripes indicates that the crack originates from a depth of 4.5 mm below the root surface of the tooth (as indicated by the arrow in Figure 2b, which is the convergence point of all the radial stripes on the fracture surface. Figure 2c,d show the scanning electron microscope (SEM) and backscatter electron (BSE) images of the inclusions (equipment is Hitachi S-3700N Scanning Electron Microscope, Japan), respectively. Bar-like defects are visible at the convergence point of the radial stripes. According to the analysis in Figure 2e, the defect area is rich in slag characteristic elements such as Al and O, suggesting that this defect may be slag entrained during the steelmaking process, leading to slag inclusion in the steel. Non-metallic inclusions in the raw materials can cause uneven stress distribution on the gear surface, leading to localized stress concentration and reducing the gear’s fatigue performance [19].

The failure analysis results indicate that alumina inclusions exist at a depth of approximately 4.5 mm beneath the surface of the gear shaft. The length of the inclusions is approximately 800 μm, and the width is about 100 μm. As evidenced by the convergence of radial crack patterns at this inclusion site (Figure 2b,c) and confirmed by EDS analysis showing high concentrations of Al and O, these inclusions acted as the primary crack initiation point. When such non-metallic inclusions (particularly oxides, silicates, and titanium nitride) are present in the raw material, the significant difference in the elastic modulus between the inclusions and the matrix leads to stress concentration at the inclusion tips and adjacent regions under cyclic loading [20]. This stress concentration promotes dislocation accumulation at the inclusion–matrix interface, facilitating fatigue crack initiation. Consequently, insufficient material purity often results in (1) cracking at inclusion sites during subsequent heat treatment due to internal stresses [21] and (2) subsurface fatigue initiation under contact stress during gear operation. When inclusions are clustered, fatigue typically initiates at inclusion tips, leading to spalling or even tooth fracture, thereby significantly reducing the gear’s fatigue life.

#### 2.1.2. Forging Process of Raw Materials

After forging, the material undergoes recrystallization treatment, which enhances plasticity and improves mechanical properties. However, if the forging process is not properly controlled, defects, such as excessive oxidation, decarburization, overheating, and internal cracks, may occur, which will significantly affect the quality control of subsequent heat treatment and the service life of the gears. For example, if not effectively removed, the decarburized layer on the surface of the material is likely to generate tensile stress on the surface after the subsequent carburization, resulting in gear cracking. When there is a relatively severe Widmanstätten structure in gear, its mechanical properties will be dramatically compromised, resulting in premature gear failure [22]. In addition, severe deformation will occur in the heat treatment process, which will cripple the precision of the tooth surface and reduce meshing accuracy, resulting in eccentric load and gear failure.

### 2.2. Effects of Heat Treatment

Due to the large load transmitted in the work process, the gear used on the track locomotive has to withstand great contact stress on the tooth surface while bearing a greater bending load at the tooth root. In the meantime, the gear is subject to enormous impact at the start or brake state. Therefore, it is required that the gear tooth surface have a high wear resistance and that the core possess a relatively high toughness, thus demanding that the heat treatment of gears in the transport industry generally use carburizing or induction hardening. Common defects in heat treatment include a substandard microstructure, unfavorable hardness of the surface or core of the block, an inappropriate depth of the hardened layer, decarburization and grain boundary oxidation on the surface, unfavorable residual stress distribution, and the presence of heat treatment cracks. The main causes are as follows:

#### 2.2.1. Surface-Hardened Layer

The surface-hardened layer’s depth significantly affects the gear’s contact fatigue and bending fatigue performance. The tooth flaking depth is related to contact stress and relative sliding velocity. The maximum depth of shear stress under contact stress is 0.786b. For the gear to have sufficient contact fatigue resistance, the depth of the layer should be twice that of the maximum shear stress. Therefore, the hardened layer should be as thick as possible to improve the gear’s anti-contact fatigue capacity [23]. However, as there is an optimum value for the bending fatigue resistance of gears, a hardened layer that is too shallow or too deep will cause the gear’s bending fatigue performance to decline. A driving gear manufactured from 20CrMnMo steel failed by cracking after only 18 months of service. The manufacturing process consisted of forging, normalizing, hobbing, carburizing, quenching (850 °C) with low-temperature tempering (610 °C), gear grinding, and shot peening. 

**Figure 3:**
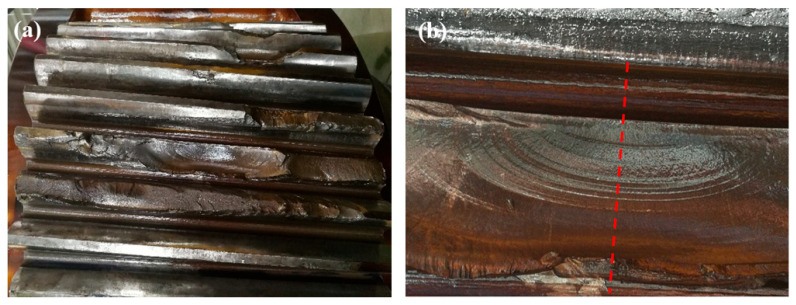
Digital camera figures of the failed gear: (**a**) tooth surface and (**b**) shell-like marks (delineated by red dashed line).

Figure 3 shows the digital camera photos of the failed gear. In Figure 3a, there is severe wear near the meshing area of each tooth surface, accompanied by traces of plastic deformation due to rolling, brittle fracture, and varying degrees of fatigue fracture. According to Figure 3b, the fatigue source of a fractured tooth is located at the tooth root. In this part, obvious shell-shaped traces are found, with typical fatigue fracture characteristics.

**Figure 4:**
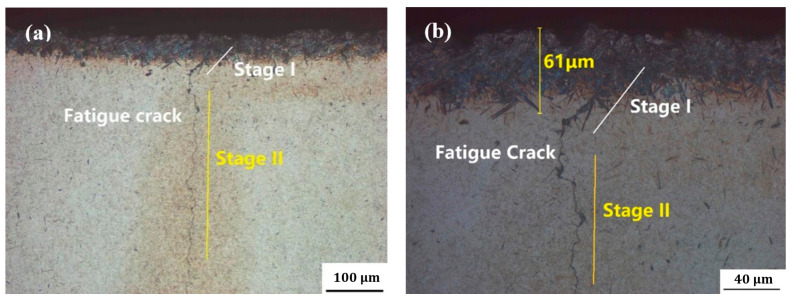
Metallographs of the cross-section near the fatigue source: (**a**) metallographic structure near the tooth root and (**b**) enlarged photo of fatigue crack at the tooth root.

Figure 4 shows the metallographs of the cross-section near the fatigue source. A fine crack of approximately 0.6 mm in length was observed near the crack initiation site, likely formed by impact during shot peening. Metallographic examination at the gear tooth root revealed a non-martensitic structural layer with a depth of approximately 60 μm, predominantly composed of coarse upper bainite. This microstructural anomaly, identified in the transition zone, fails to meet the microstructural integrity requirements specified for the hardened case in GB/T 25744—2010 [23].

The failure analysis results indicate that the faulty gearbox initially exhibited fatigue folding at the tooth root. Testing revealed the presence of a non-martensitic structure approximately 60 μm deep in the tooth root region. The appearance of this non-martensitic structure suggests incomplete transformation into the ideal martensitic microstructure during the post-carburizing quenching process, resulting in an insufficiently hardened case. This abnormal microstructure exacerbates stress concentration at the tooth root and deteriorates the residual stress distribution [24].

Furthermore, insufficient cooling rates during quenching (excessively high oil temperatures or inadequate agitation) can lead to the transformation of austenite into coarse upper bainite, which exhibits high brittleness and tends to serve as a crack propagation pathway. Coarse upper bainite was indeed observed in the carburized transition layer of this gear. The combined effects of these detrimental factors caused hardened layer defects that significantly reduced the gear’s bending fatigue life, rendering it incapable of meeting service requirements under high-load impacts. Consequently, when subjected to substantial bending stresses during operation, the gear became highly susceptible to premature fatigue fracture.

#### 2.2.2. Overheating

Overheating during the heat treatment process can lead to coarse austenite grains. When cooled at an appropriate rate (air cooling), a Widmanstätten structure may form in the gear material, significantly reducing its mechanical properties (such as tensile strength and fatigue limit) and leading to premature failure [25]. If the heating temperature is excessively high, causing severe coarsening of austenite grains, and non-equilibrium cooling occurs, both the Widmanstätten structure and the coarse upper bainite may coexist, further exacerbating the detrimental effects on the material’s mechanical performance.

#### 2.2.3. Over-Deformation of the Gear

During improper heat treatment, uncontrolled excessive deformation of the gear geometry occurs due to temperature gradients, phase transformation stresses, or uneven residual stress distribution. This affects subsequent grinding allowance control and product accuracy, leading to tooth surface distortion [26]. When the gear meshes, the load distribution along the tooth width becomes uneven, resulting in non-uniform wear on the tooth’s surface. Overloaded areas may experience fatigue spalling or even creasing and tooth breakage. Excessive deformation of the gear is one of the factors contributing to premature gear failure.

### 2.3. Effects of Manufacturing Processes

In order to manufacture gears with high geometric accuracy, the tooth surfaces need to undergo grinding treatment. However, the grinding process of gears that have undergone surface hardening treatment may result in defects such as grinding cracks and grinding burns. Moreover, defective gears are prone to seizing and falling off during aging, thereby causing a decline in fatigue performance. Due to the changes in microstructure during the aging process, there are differences between the local and overall performance, leading to local stress concentration when the components are loaded (especially under high-speed and heavy-load conditions), which in turn triggers microcracks and ultimately results in damage to the entire component.

In addition, the gear tooth’s machining accuracy is not enough. Too much tooth orientation error will cause eccentric load in the tooth direction; pitch deviation and tooth shape error will cause undesirable tooth height contact, and tooth surface roughness is likely to cause pitting or scuffing.

**Figure 5:**
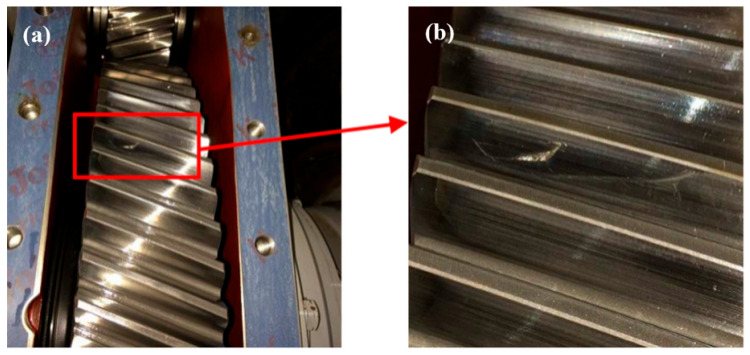
Digital camera photos of the driven gear in a high-speed train: (**a**) fracture and (**b**) enlarged photo of the fracture.

Figure 5 shows the digital camera photo of a tooth flaw in the driven gear of a certain type of high-speed train. The gear surface was heat-treated with induction hardening, but after two years of service, an arcuate crack was found on the tooth surface, with the local existence of peeling pits. The crack is about 15 mm long, crossing obliquely with the tooth surface processing marks, where there are slight burrs on touch. In addition, no abnormalities are found on both sides of the crack, except for the metalescence.

**Figure 6:**
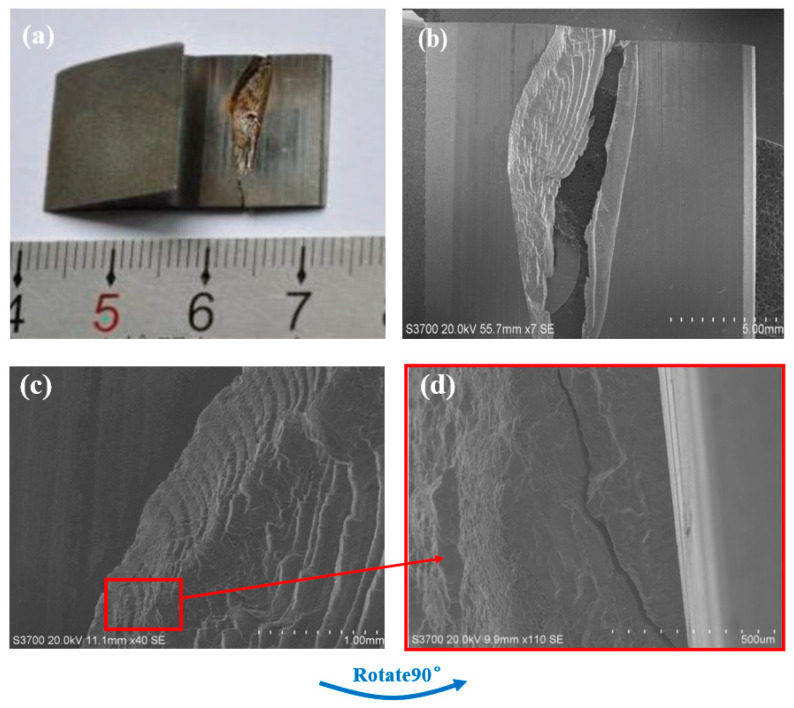
(**a**) Low-magnification digital camera photograph of the cross-section and (**b**–**d**) SEM images of the cross-section.

Figure 6 shows the digital camera photograph and SEM images of the cross-section, which was prepared by cutting the gear parallel to the tooth surface, extracting the crack region, and manually fracturing it. As shown in Figure 6a,b, the cross-section can be categorized into near-surface and near-center regions. Clearly visible beach marks are observed in the near-surface area. The convergence direction of these beach marks indicates that the fatigue origin is located within the boxed region, approximately 0.15 mm below the surface. Figure 6d displays the SEM image of the fatigue origin area after rotation, where no raw material defects, such as porosity or slag inclusions, are detected. These characteristics confirm that the tooth flank failure is a fatigue-induced spalling crack. The subsurface location of the crack initiation site is consistent with the theoretical maximum contact stress zone during gear meshing.

**Figure 7:**
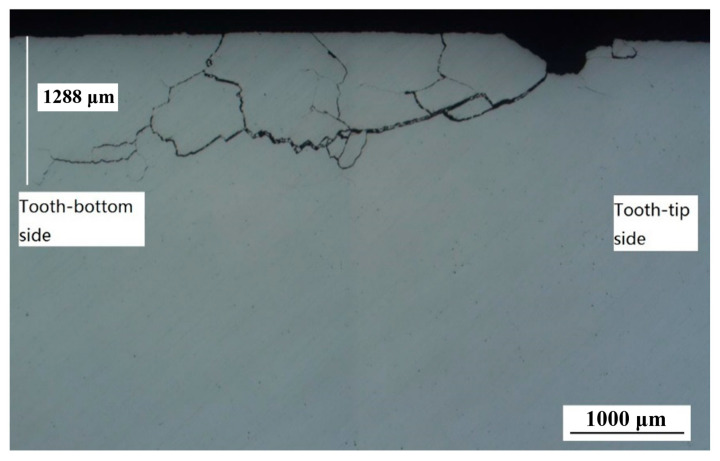
Metallographs of the cross-section.

**Figure 8:**
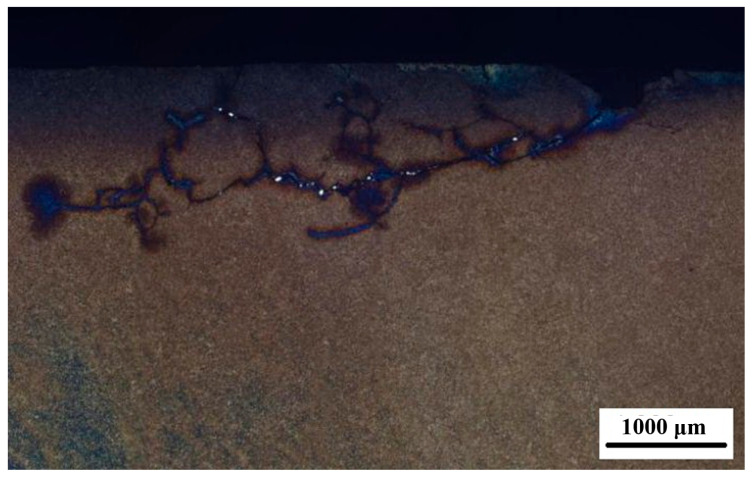
Metallographs on both sides of the crack after etching.

As shown in Figure 7, the crack exhibits a wide opening and propagates at an angle of approximately 45° to the surface, terminating in a thin upturned tail. Three branching cracks in the central region extend toward the surface, displaying characteristic features of contact fatigue. Figure 8 shows the metallographs from both sides of the crack after etching with nitric acid ethanol solution. Crescent-shaped ‘dark spots’ are visible in the tooth flanks adjacent to the defects. These ‘dark spots’ show greater susceptibility to etching compared to the surrounding material. The microstructure in these regions consists of middle-tempered martensite with dispersed granular carbides. The precipitation of these carbides likely resulted from the decomposition of retained austenite during the secondary tempering stage, indicating that the ‘dark spot’ areas experienced thermal exposure, with temperatures reaching the tempering range of 350 °C to 500 °C.

**Figure 9:**
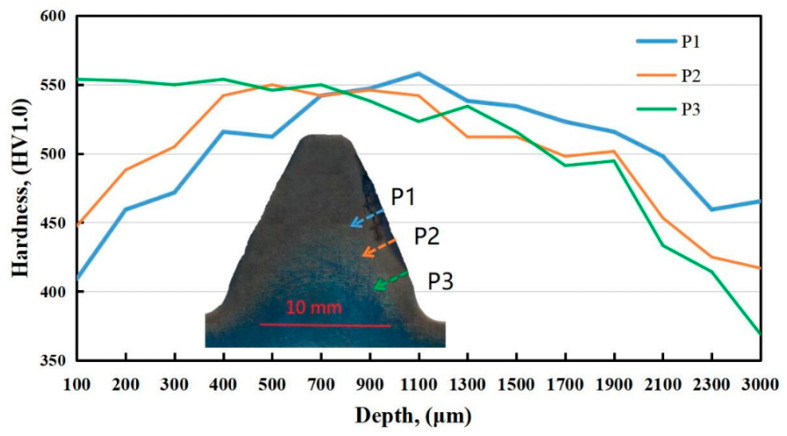
Hardness curve of the middle area (P1), edge area (P2) of the black spot, and normal area (P3).

In Figure 9, the results of hardness gradient tests on areas in the middle, on the edges, and in the vicinity of the ‘black spots’ (1 to 3) show that the hardness of the spot is relatively low and that the hardness value of the ‘black spot’ increases with the depth into the surface. Based on these features, it can be further speculated that the spot is a temper burn zone, with a certain temperature gradient when "black spots" are formed.

The failure analysis results indicate that localized overheating during the grinding process on the driven gear’s surface led to temper softening, forming crescent-shaped ’black spots.’ The lower the hardness in these black spot regions, the lower the surface stress state, which is identified as the primary cause of fatigue-induced spalling cracks on the tooth surface. To achieve high geometric precision in gears, grinding of the tooth surface is essential. However, the grinding process for surface-hardened gears is prone to defects such as grinding cracks and grinding burns. Additionally, defective gears are susceptible to seizing and spalling during the run-in phase, resulting in reduced fatigue performance. Due to microstructural changes in the burn-affected zones, localized properties diverge from the bulk material, creating stress concentrations under load (particularly in high-speed and heavy-duty conditions). This initiates microcracks and ultimately leads to component failure.

### 2.4. Effects of Operational Factors 

**Figure 10:**
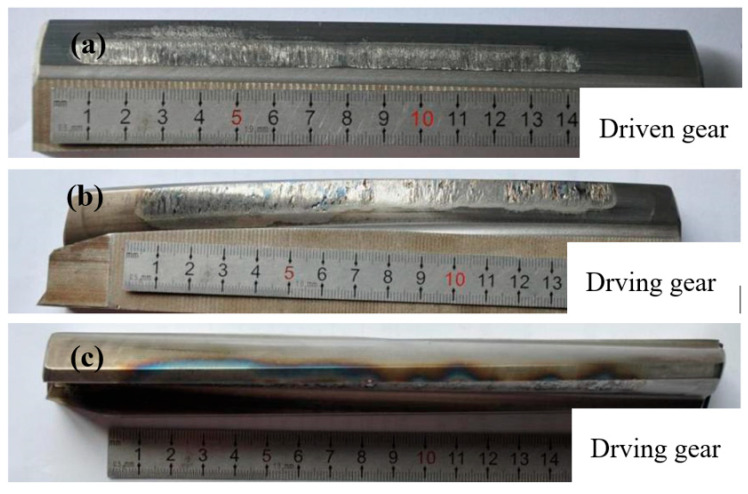
Digital camera photos of the driven and driving gear: (**a**) tooth surface of the driven gear, (**b**) tooth surface of the driving gear, and (**c**) tooth tip of the driving gear.

**Figure 11:**
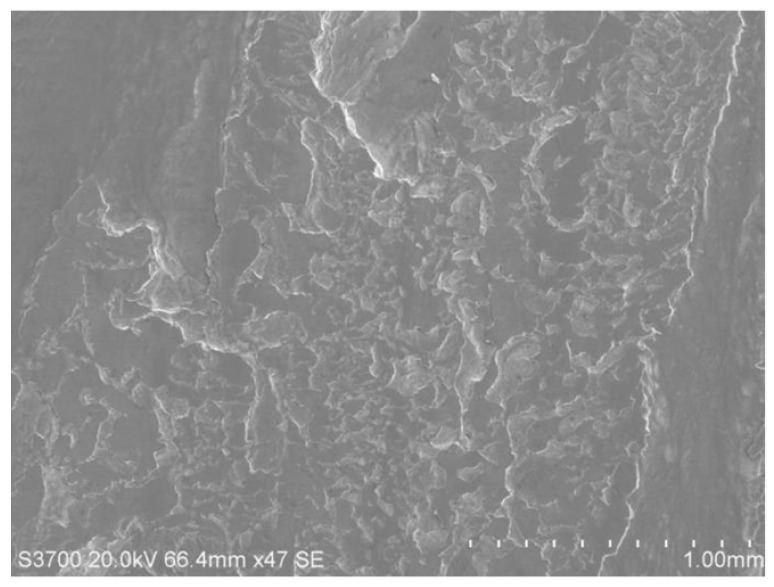
SEM image of the damage area on the tooth surface.

Figure 10 shows the digital camera photos of a type of locomotive’s gear tooth’s flank surface. The mating surfaces of the driving and driven gears are damaged, leading to a belt-shaped rough portion. The top of the driving gear tooth is blue and tempered in color, showing the characteristics of hot scuffing. Figure 11 shows the SEM image of tooth surface damage of the driving gear, showing that the microscopic damage is caused by adhesive wear.

**Figure 12:**
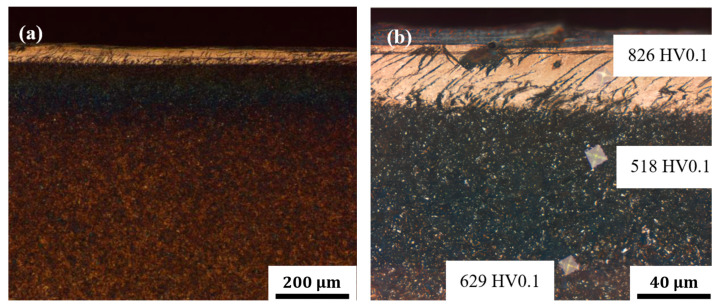
Metallographs of the cross-section: (**a**) metallographic structure near the damage zone and (**b**) enlarged photo of the surface layer.

Figure 12 shows metallographs of the cross-section near the damage zone. The cross-section can be divided into three regions based on the color difference. As shown in Figure 12b, the white surface structure is secondary quenched martensite, which is not easily corroded and has a relatively high hardness (826 HV); the subsurface black tissue is composed of high- and middle-tempered martensite formed after tempering, which is easily corroded and has a lower hardness than the substrate (518 HV); and the brown matrix color is the tempered martensite, with a hardness of 629 HV. The microstructure characteristics mentioned above indicate that the tooth surface suffered from a lot of heat during gear service.

The failure analysis results indicate that the damage morphology of the mating surfaces of the driving and driven gears conforms to the characteristics of thermal scuffing. The failure mechanism is attributed to insufficient lubrication supply, which leads to overheating in the gear meshing zone, rupture of the oil film, and subsequent metal adhesion, ultimately resulting in gear failure. Regarding the impact of operational factors, a major gear manufacturer conducted a statistical analysis of 931 gear failure cases between 1930 and 1975. From the perspective of failure causes, the results show that the vast majority of failures (74.7%) were related to operational factors, including poor lubrication, foreign object contamination, improper assembly, impact overload, and environmental conditions. In the rail transportation industry, gears typically operate in lubricated environments, where lubrication conditions significantly influence gear reliability and service life. If the lubrication system design is flawed, or if the lubrication method, lubricant performance, or oil volume control are improperly managed, issues such as cuffing, overheating, and excessive wear may occur on the gear tooth surfaces.

## 3. Conclusions and Recommendations

The analysis of gear failures in the rail transit system indicates that failures are usually caused by synergistic interactions of multiple factors, including material defects, improper heat treatment, manufacturing process flaws, and in-service state, etc. The principal failure mechanisms and their corresponding countermeasures, derived from specific case evidence, are summarized as follows:

1. **Material Defects**: In the first failure case of the gear shaft, the quenching crack initiated from an alumina inclusion (~800 µm long and ~100 µm wide) located approximately 4.5 mm beneath the surface. Such non-metallic inclusions act as stress concentrators under cyclic loading, initiating fatigue cracks. To mitigate this, we recommend implementing ultrasonic testing for incoming material inspection and enforcing stricter inclusion rating controls, particularly for high-stress component regions.
2. **Improper Heat Treatment**: The analysis of a fractured driving gear revealed a non-martensitic microstructure (approximately 60 µm deep, containing coarse upper bainite) at the tooth root. This microstructural anomaly, resulting from an insufficient quenching cooling rate, significantly reduced bending fatigue strength and promoted crack initiation. The corrective measure involves utilizing multi-zone controlled-atmosphere quenching furnaces with real-time monitoring of oil temperature and agitation intensity to ensure optimal cooling rates. Post-process validation via residual stress analysis (e.g., X-ray diffraction) is also recommended.
3. **Grinding-Induced Defects**: The driven gear failure was characterized by crescent-shaped ’black spots’ identified as temper burns. These areas exhibited substantial hardness reduction (by ~100 HV), transforming them into initiation sites for contact fatigue spalling. To prevent this, we propose integrating real-time process monitoring, including infrared thermography and acoustic emission, during gear grinding. Furthermore, optimizing grinding wheel parameters and coolant application strategies is essential to avoid localized overheating.
4. **Insufficient Lubrication**: Several failure cases exhibited thermal scuffing and adhesive wear on tooth flanks, directly attributable to inadequate lubrication or oil film breakdown. This aligns with industry statistics indicating that lubrication issues contribute to nearly 75% of gear failures. Countermeasures include optimizing lubrication system design (nozzle positioning and oil flow), selecting high-performance extreme-pressure lubricants, and implementing online monitoring of oil cleanliness and moisture content to ensure consistent lubrication integrity.

In summary, the reliability of rail transit gears is contingent upon comprehensive quality control across the entire lifecycle—from material selection and heat treatment to manufacturing and in-service monitoring. The findings from these failure cases underscore the necessity of root cause analysis based on specific failure modes and empirical evidence. Future efforts should focus on integrating advanced non-destructive evaluation techniques and optimized, controlled manufacturing processes to proactively mitigate these prevalent failure modes and enhance gear longevity.

## Data Availability

The original contributions presented in this study are included in the article. Further inquiries can be directed to the corresponding author.
